# A Bibliometric Analysis of the Most Cited Journal Articles in Kidney Transplantation

**DOI:** 10.7759/cureus.38104

**Published:** 2023-04-25

**Authors:** Badi Rawashdeh, Mohammad AbuAssi, Yazan Al-Adwan, Ashraf El-Hinnawi

**Affiliations:** 1 Department of Transplant, Medical College of Wisconsin, Milwaukee, USA; 2 Department of Anesthesia and Critical Care, Jordan Hospital, Amman, JOR; 3 Division of Transplant Surgery, University of Virginia, Charlottesville, USA; 4 Division of Transplant Surgery, University of Florida, Gainesville, USA

**Keywords:** countries, journals, authors, research, bibliometric analysis, kidney transplantation

## Abstract

Citation analysis uses the number of times an author, article, or publication has been cited to determine its relative importance or effect. To provide an overview and identify the articles that have gotten the most attention in the field of kidney transplantation, this bibliometric analysis was conducted to analyze the top 100 most cited articles in the Scopus database. The search terms "kidney" and "renal" and transplant-related words such as "transplant," "donor," "recipient," and "procurement" were used to search the Scopus database. Articles up to the query date of December 21, 2022, were included, and all document types including articles, reviews, conference papers, editorials, book chapters, and meeting abstracts were analyzed. The analysis focused on authors, annual trends, journals, and countries. A total of 68,271 articles related to kidney transplantation were published in the Scopus database up to the search date of December 21, 2022. The top 100 cited papers had a total of 76,029 citations, with a mean citation count of 760.3 ± 284.6. The most cited article was a clinical practice guideline paper published by the Kidney Disease: Improving Global Outcomes (KDIGO) Work Group. The top cited journals were the New England Journal of Medicine, Transplantation, and the American Journal of Transplantation. The most productive authors were primarily based in the United States, with the most frequently cited first author being Kasiske B.L. The greatest number of articles and citations were published between 2000 and 2005. This bibliometric analysis provides a comprehensive overview of the top cited articles in the field of kidney transplantation. The results highlight the most influential and impactful research, as well as the most productive authors, journals, and countries. These findings can be used to guide future research and support decision-making in funding and policy.

## Introduction and background

Kidney transplantation is a crucial procedure for patients with end-stage renal disease (ESRD) that provides a survival advantage over maintenance on dialysis [1]. The field of kidney transplantation has undergone substantial advancements in surgical techniques, immunosuppression, and patient outcomes. Major developments in transplantation and immunology have expanded the pool of acceptable donors and recipients, while improved organ matching and preservation, combined with the advancements in surgical protocols and chemoprophylaxis, have all contributed to improved clinical outcomes [2,3].

Evaluative bibliometrics is a branch of quantitative science that measures the efficacy of a study using techniques such as citation analysis [4]. The frequency with which an article has been cited allows us to measure the effect of an article over time. Highly cited articles demonstrated the importance of the subjects discussed in these articles among the scientific community of the relevant medical discipline. Bibliometric analysis can be helpful in providing an overview of the publications that have the most influence in certain areas of medical practice, as well as providing an idea of the most influential institutions and authors to stimulate collaborations [5].

Furthermore, bibliometric analysis is a valuable tool for evaluating the impact and trends in a specific field of research [6]. In the context of kidney transplantation, the analysis of the top 100 cited articles provides valuable insights into the most influential studies and authors in this field [7,8].

To determine the top 100 kidney transplantation articles cited most frequently across all journals, we used bibliometric analysis. In this analysis, we focused on authors, annual trends, journals, and countries to gain insights into the most influential and impactful research in the field.

## Review

Materials and methods

Data Collection and Retrieval Methods

We searched the Scopus database using the terms "kidney" and "renal," as well as transplant-related words such as "transplant," "donor," "recipient," and "procurement." The terms were searched in the title/abstract. The exact algorithm was as follows: TITLE-ABS-KEY ("kidney transplant" OR "renal transplant" OR "kidney donor" OR "renal donor" OR "kidney recipient*" OR "kidney procurement"). All articles up to the query date on December 21, 2022, were included. Additionally, we included all document types, such as articles, reviews, conference papers, editorials, book chapters, and meeting abstracts. Articles unrelated to the field of transplantation were excluded. Articles with missing information were manually removed from the spreadsheet. All included articles were in the English language. A list of the top 100 cited articles was created.

Data Analysis

All analyses were citation count-based. We analyzed authors, annual trends, journals, and countries. Tables and figures were generated using VOSviewer version 1.6.18 and Microsoft Excel from Office 365 (Microsoft Corp., Redmond, WA, USA). A flowchart was structured to demonstrate included/excluded results. Continuous data were reported as mean ± standard deviation (SD), while frequencies were reported as numbers (n). We compared mean citations between different subgroups using the analysis of variance (ANOVA) test; in case of violating the ANOVA test assumptions, we used the nonparametric Kruskal-Wallis test. A p-value of 0.05 or less was deemed statistically significant. All statistical analyses were done using R statistical language (Vienna, Austria).

Results

**Figure 1:**
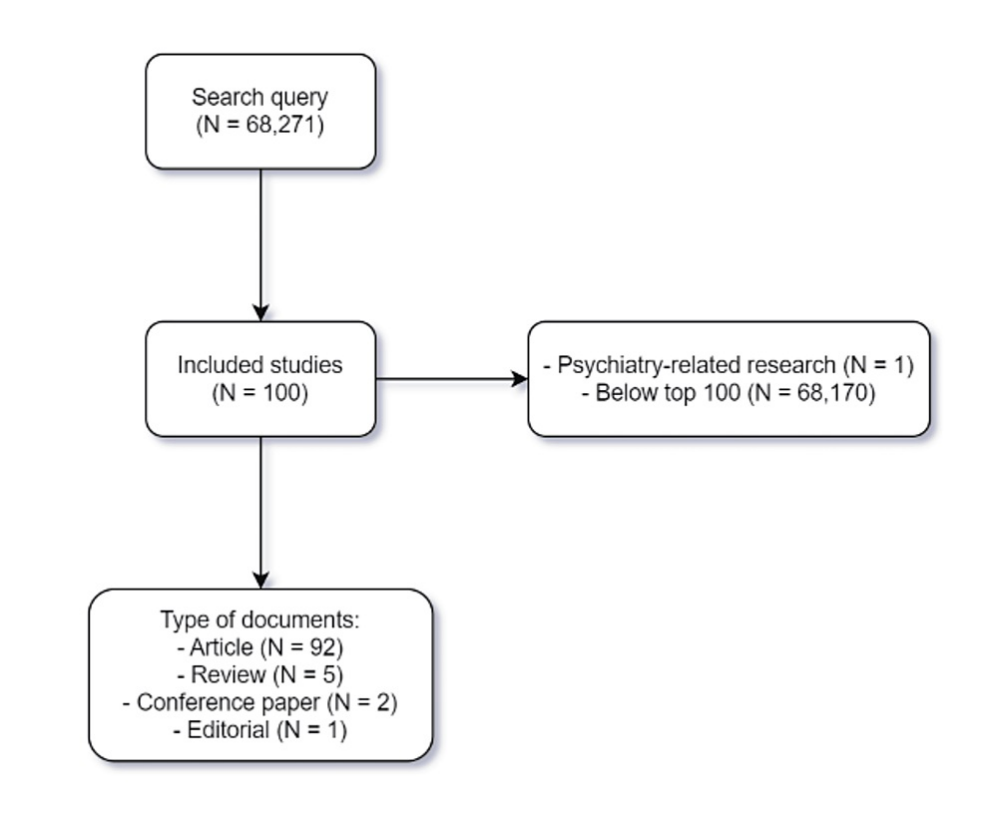
Flowchart of search results and types of included documents

Up to the search date, a total of 68,271 published articles were related to kidney transplantation in the Scopus database. The top 100 cited papers were included, but one study was excluded due to being psychiatry-related. As for the document type, 92 studies were articles, five studies were reviews, two were conference papers, and one study was editorial (Figure 1). The 100 articles have cited a total of 76,029 times, with a mean ± SD of 760.3 ± 284.6.

Over 910 authors were included in our analysis, 83 of which were the first authors on at least one document. When the first authors on the documents were assessed, Kasiske B.L. was the first author of five articles accounting for 3,122 citations. Opelz G. appeared on four documents with 2,948 citations. Vincenti F., Ojo A.O., and Meier-Kriesche H.-U. each were the first authors of three documents (n = 2,281, 2,204, and 2,093, respectively). Table 1 demonstrates a list of the top cited authors from the included studies.

**Table 1:** First authors of the 100 most cited articles in the field of kidney transplantation *The authors' total number of citations within the included top 100 articles KDIGO: Kidney Disease: Improving Global Outcomes, CKD-MBD: chronic kidney disease-mineral and bone disorder

ID	Name	Number of documents	Total citations*
1	Kasiske B.L.	4	3,122
2	Opelz G.	4	2,948
3	Vincenti F.	3	2,281
4	Ojo A.O.	3	2,204
5	Meier-Kriesche H.-U.	3	2,093
6	Hariharan S.	2	2,232
7	Terasaki P.I.	2	1,984
8	Hirsch H.H.	2	1,779
9	Port F.K.	2	1,235
10	Brennan D.C.	2	1,140
11	KDIGO CKD-MBD Work Group	1	2,053
12	KDIGO Transplant Work Group	1	1,783
13	Nankivell B.J.	1	1,653
14	Ekberg H.	1	1,422
15	Cho S.	1	1,286
16	Solez K.	1	1,285
17	Sellarés J.	1	1,065
18	Pappas P.G.	1	1,035
19	Kamar N.	1	1,024
20	Pirsch J.D.	1	1,019
21	Fioretto P.	1	1,011
22	Engels E.A.	1	969
23	Kestenbaum B.	1	945
24	Nortier J.L.	1	923
25	Rule A.D.	1	906
26	Ratner L.E.	1	869
27	Groth C.G.	1	854
28	Kawai T.	1	850
29	Stallone G.	1	819
30	Ibrahim H.N.	1	816
31	Herzog C.A.	1	801
32	Holdaas H.	1	793
33	Perico N.	1	757
34	Hirsch J.S.	1	753
35	Moers C.	1	742
36	Uchino S.	1	729
37	Bullingham R.E.S.	1	718
38	Wiebe C.	1	713
39	Ortho Multicenter Transplant Study Group	1	710
40	Jensen P.	1	707
41	Kootstra G.	1	692
42	Noris M.	1	686
43	Lown K.S.	1	680
44	Lamb K.E.	1	672
45	Dragun D.	1	670
46	Nashan B.	1	669
47	Rao P.S.	1	666
48	Muzaale A.D.	1	657
49	Mcmurray J.J.V.	1	654
50	Loupy A.	1	637
51	Almond P.S.	1	636
52	Mayer A.D.	1	633
53	Lefaucheur C.	1	620
54	Hesselink D.A.	1	617
55	El-Zoghby Z.M.	1	611
56	Schortgen F.	1	599
57	Poggio E.D.	1	593
58	Changelian P.S.	1	588
59	Czock D.	1	585
60	Macdonald A.S.	1	581
61	Poulsom R.	1	581
62	Najarian J.S.	1	561
63	Newell K.A.	1	552
64	Kauffman H.M.	1	549
65	Reeve C.E.	1	549
66	Segev D.L.	1	548
67	Palacios G.	1	544
68	Vo A.A.	1	544
69	Merion R.M.	1	543
70	Barton F.B.	1	540
71	Mjøen G.	1	537
72	Einecke G.	1	531
73	Yarlagadda S.G.	1	531
74	Kronbach T.	1	529
75	Laterza O.F.	1	521
76	Montgomery R.A.	1	521
77	Birkeland S.A.	1	517
78	Hoover R.	1	516
79	Kinlen L.J.	1	515
80	Metzger R.A.	1	512
81	Yamada K.	1	510
82	Pereira M.R.	1	503
83	Ketteler M.	1	501

**Figure 2:**
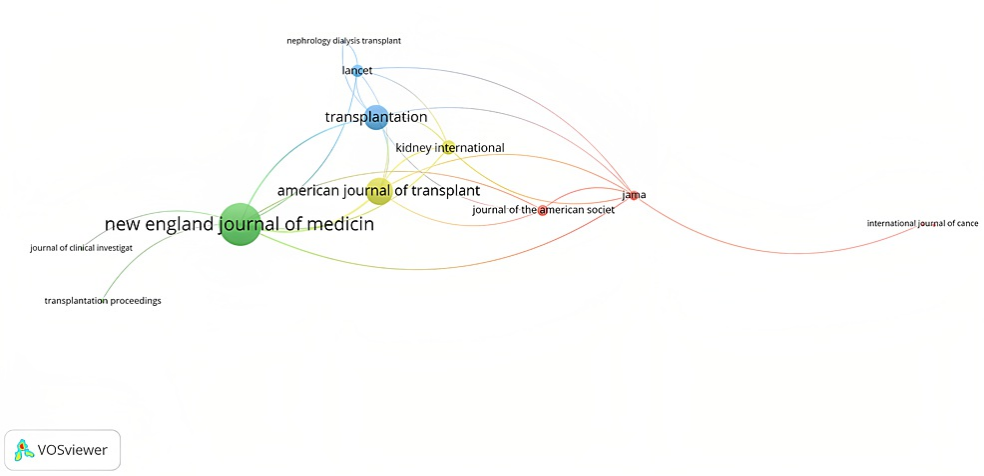
Visualization of the most cited journals

The most frequently published journals were the New England Journal of Medicine (n = 22), Transplantation (n = 14), and the American Journal of Transplantation (n = 14). The New England Journal of Medicine had the highest number of citations (n = 19,528), followed by the American Journal of Transplantation (n = 11,392) and then Transplantation (n = 10,482) (Table 2). Figure 2 visualizes the most cited journals and the interconnections between them.

**Table 2:** Bivariate analysis of mean citations by article's characteristics SD: standard deviation

	Subgroup	Number	Total number of citations	Total number of citations (mean ± SD)	p-value
Article year	<2000	30	22,740	585.7 ± 215.1	0.468
	2000-2005	37	27,501	743.3 ± 266.9	
	2006-2010	21	17,587	837.5 ± 422.1	
	2011-2015	9	6,444	716 ± 181.8	
	2016-2020	3	1,757	628 ± 144.9	
Authors' country	United States	57	41,066	720.5 ± 217.9	0.139
	Germany	8	5,373	671.6 ± 149.9	
	Canada	6	4,724	787.3 ± 314.8	
	United Kingdom	4	2,383	595.8 ± 62	
	France	4	2,880	720 ± 203.3	
	Italy	4	3,273	818.3 ± 139.5	
	Norway	3	2,037	679 ± 130.3	
	Netherlands	3	2,051	683.7 ± 62.9	
	Switzerland	3	2,308	769.3 ± 225	
	Sweden	2	2,276	1,138 ± 401.6	
	Global	2	3,836	1,918 ± 190.9	
	Australia	1	1,653	-	
	Belgium	1	923	-	
	Denmark	1	517	-	
	Japan	1	729	-	
Document type	Article	92	70,583	767.2 ± 293.19	0.790
	Review	5	3,588	717.6 ± 163.3	
	Conference paper	2	1,204	602 ± 127.3	
	Editorial	1	654	-	
Journals	New England Journal of Medicine	22	19,528	887.6 ± 318.6	0.172
	American Journal of Transplantation	14	11,392	813.7 ± 340.3	
	Transplantation	14	10,482	748.7 ± 212.5	
	Kidney International	8	5,428	678.5 ± 260	
	Lancet	7	4,506	643.7 ± 101.6	
	Journal of the American Society of Nephrology	6	4,061	676.8 ± 137.1	
	JAMA	5	3,328	665.6 ± 176	
	Clinical Pharmacology and Therapeutics	3	1,826	608.7 ± 75.8	
	Clinical Pharmacokinetics	2	1,303	651.5 ± 94	
	Kidney International Supplements	2	2.707	1,353.5 ± 989.2	
	Transplantation Proceedings	2	1,629	814.5 ± 173.2	
	American Journal of Clinical Pathology	1	921	-	
	Annals of Internal Medicine	1	906	-	
	British Medical Journal	1	515	-	
	Clinical Chemistry	1	521	-	
	Clinical Infectious Diseases	1	1,035	-	
	Clinical Journal of the American Society of Nephrology	1	686	-	
	Critical Care Medicine	1	729	-	
	Diabetes Care	1	540	-	
	International Journal of Cancer	1	517	-	
	Journal of Clinical Investigation	1	552	-	
	Journal of Pathology	1	581	-	
	Journal of the American Academy of Dermatology	1	707	-	
	Nature Medicine	1	510	-	
	Nephrology Dialysis Transplantation	1	531	-	
	Science	1	588	-	

**Figure 3:**
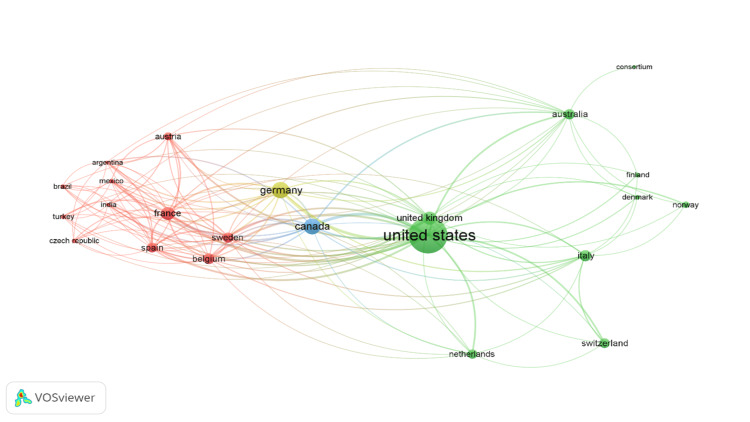
Visualization of authors' countries

The first authors' institutes were assessed, 57 of which were from the United States with the highest citations (n = 41,066). The second most frequent country was Germany (n = 8) with 5,373 citations, followed by Canada (n = 6) with 4,724 citations (Figure 3).

The greatest number of articles were published from 2000 to 2005 (n = 37). Thirty articles were published before the year 2000, and 21 articles were published from 2006 to 2010. Accordingly, most citations were from 2000 to 2005 (n = 27,501), followed by the period before the year 2000 (n = 22,740).

Analysis of the top 100 most cited articles according to year revealed that there was no significant difference in mean citations between year groups (p = 0.468). Furthermore, no significant differences were found between authors' countries, document types, or journals in terms of the mean number of citations (p = 0.139, 0.790, and 0.172, respectively) (Table 2).

The top 100 cited articles in the kidney transplantation field are shown in the Appendices. “KDIGO clinical practice guidelines for the prevention, diagnosis, evaluation, and treatment of Chronic Kidney Disease-Mineral and Bone Disorder (CKD-MBD)” published by the Kidney Disease: Improving Global Outcomes “Chronic Kidney Disease-Mineral and Bone Disorder “ (KDIGO CKD-MBD) Work Group, which was published in the Kidney International Supplements journal, was the most cited study (n = 2,053) [9], followed by a guideline paper published in the American Journal of Transplantation, also done by the KDIGO Work Group (n = 1,783) [10]. The least cited article had 501 citations published by Ketteler et al. [11] in Kidney International.

Discussion

Bibliometric analysis and visualization tools, such as VOSviewer, have gained prominence for their ability to assess and present complex citation patterns and research trends in various fields [12]. These tools enable researchers, clinicians, and decision-makers to identify influential publications, emerging research topics, and leading contributors within the domain [13,14]. The field of kidney transplantation has seen significant advancements in recent years, leading to improved long-term allografts and patient survival. The introduction of cyclosporine in 1983 marked a major turning point in the history of solid-organ transplantation, significantly reducing the risk of rejection and leading to a proportional increase in one-year patient and graft survival rate for a kidney transplantation to over 90% [3,15].

A previous bibliometric analysis of the most-cited kidney transplantation papers made use of the Web of Science database, covering articles up to 2019 [16]. In contrast, our study employs the Scopus database and encompasses a more diverse range of document types, resulting in a broader and more current assessment of kidney transplantation literature. By including articles, reviews, conference papers, editorials, book chapters, and meeting abstracts through December 21, 2022, our research captures the latest and most influential publications in the field. Furthermore, our study delves deeper into the relationships between citations and factors such as publication year, authors' countries, document types, and journals, shedding light on emerging trends and citation patterns within kidney transplantation research. This comprehensive approach provides valuable insights that were not present in the earlier study, making our analysis a superior resource for researchers, clinicians, and decision-makers in the field of kidney transplantation.

Kidney transplantation had more publications than any other organ transplantation specialty [17]. The top 100 cited articles provide a snapshot of the most influential and impactful research in the field. The results show that the most cited article was a clinical practice guideline paper published by the Kidney Disease: Improving Global Outcomes (KDIGO) Work Group, which highlights the importance of guidelines and best practices in the field of kidney transplantation. The findings also indicate that the most productive authors were primarily from the United States, with the most frequently cited first author being Kasiske B.L. The top cited journals were the New England Journal of Medicine, Transplantation, and the American Journal of Transplantation, which suggests that these journals are at the forefront of research in the field of kidney transplantation. It is worth noting that the greatest number of articles and citations were published between 2000 and 2005, which suggests that there has been a significant increase in research in the field of kidney transplantation over the past two decades. However, the results also showed that there was no significant difference in mean citations between year groups, authors' countries, document types, or journals. This suggests that while the field of kidney transplantation has seen significant progress, there is still room for improvement, and further research is needed to advance the field.

## Conclusions

This bibliometric analysis provides valuable insights into the current state of research in the field of kidney transplantation. The results highlight the most influential and impactful research in the field, as well as the most productive authors, journals, and countries. The findings of this study can be used to guide future research and support decision-making in funding and policy.

## Appendices

Table 3 presents the top 100 cited articles in the kidney transplantation field.

**Table 3:** The 100 most cited articles in the field of kidney transplantation

ID	Title	Year	Authors	Cited by
1	KDIGO clinical practice guidelines for the prevention, diagnosis, evaluation, and treatment of Chronic Kidney Disease-Mineral and Bone Disorder (CKD-MBD)	2009	Kidney Disease: Improving Global Outcomes (KDIGO) CKD-MBD Work Group	2,053
2	KDIGO clinical practice guideline for the care of kidney transplant recipients	2009	Kidney Disease: Improving Global Outcomes (KDIGO) Transplant Work Group	1,783
3	The natural history of chronic allograft nephropathy	2003	Nankivell B.J., Borrows R.J., Fung C.L.-S., O'Connell P.J., Allen R.D.M., et al.	1,653
4	Improved graft survival after renal transplantation in the United States, 1988 to 1996	2000	Hariharan S., Johnson C.P., Bresnahan B.A., Taranto S.E., McIntosh M.J., et al.	1,622
5	Reduced exposure to calcineurin inhibitors in renal transplantation	2007	Ekberg H., Tedesco-Silva H., Demirbas A., Vítko Š., Nashan B., et al.	1,422
6	Mycophenolate mofetil for the prevention of acute rejection in primary cadaveric renal allograft recipients	1995	Cho S., Danovitch G., Deierhoi M., Ferguson R., Gonwa T., et al.	1,286
7	International standardization of criteria for the histologic diagnosis of renal allograft rejection: the Banff working classification of kidney transplant pathology	1993	Solez K., Axelsen R.A., Benediktsson H., Burdick J.F., Cohen A.H, et al.	1,285
8	Understanding the causes of kidney transplant failure: the dominant role of antibody-mediated rejection and nonadherence	2012	Sellarés J., De Freitas D.G., Mengel M., Reeve J., Einecke G., et al.	1,065
9	High survival rates of kidney transplants from spousal and living unrelated donors	1995	Terasaki P.I., Cecka J.M., Gjertson D.W., Takemoto S.	1,063
10	Diabetes mellitus after kidney transplantation in the United States	2003	Kasiske B.L., Snyder J.J., Gilbertson D., Matas A.J.	1,054
11	Invasive fungal infections among organ transplant recipients: results of the transplant-associated infection surveillance network (Transnet)	2010	Pappas P.G., Alexander B.D., Andes D.R., Hadley S., Kauffman C.A., et al.	1,035
12	Hepatitis E virus and chronic hepatitis in organ-transplant recipients	2008	Kamar N., Selves J., Mansuy J.-M., Ouezzani L., Péron J.-M., et al.	1,024
13	A comparison of tacrolimus (FK506) and cyclosporine for immunosuppression after cadaveric renal transplantation	1997	Pirsch J.D., Miller J., Deierhoi M.H., Vincenti F., Filo R.S.	1,019
14	Reversal of lesions of diabetic nephropathy after pancreas transplantation	1998	Fioretto P., Steffes M.W., Sutherland D.E.R., Goetz F.C., Mauer M.	1,011
15	Prospective study of polyomavirus type BK replication and nephropathy in renal-transplant recipients	2002	Hirsch H.H., Knowles W., Dickenmann M., Passweg J., Klimkait T., et al.	975
16	Lack of improvement in renal allograft survival despite a marked decrease in acute rejection rates over the most recent era	2004	Meier-Kriesche H.-U., Schold J.D., Srinivas T.R., Kaplan B.	974
17	Spectrum of cancer risk among US solid organ transplant recipients	2011	Engels E.A., Pfeiffer R.M., Fraumeni J.F., Jr., Kasiske B.L., Israni A.K., et al.	969
18	Serum phosphate levels and mortality risk among people with chronic kidney disease	2005	Kestenbaum B., Sampson J.N., Rudser K.D., Patterson D.J., Seliger S.L., et al.	945
19	Effect of blood transfusions on subsequent kidney transplants	1973	Opelz G., Sengar D.P., Mickey M.R., Terasaki P.I.	937
20	Urothelial carcinoma associated with the use of a Chinese herb (Aristolochia fangchi)	2000	Nortier J.L., Martinez M.-C.M., Schmeiser H.H., Arlt V.M., Bieler C.A., et al.	923
21	Microdroplet testing for HLA-A, -B, -C, -D antigens. The Philip Levine Award lecture	1978	Terasaki P.I., Bernoco D., Sik Park M., Ozturk G., Iwaki Y.	921
22	Using serum creatinine to estimate glomerular filtration rate: accuracy in good health and in chronic kidney disease	2004	Rule A.D., Larson T.S., Bergstralh E.J., Slezak J.M., Jacobsen S.J., Cosio F.G.	906
23	Laparoscopic live donor nephrectomy	1995	Ratner L.E., Ciseck L.J., Moore R.G., Cigarroa F.G., Kaufman H.S., Kavoussi L.R.	869
24	Cancer after kidney transplantation in the United States	2004	Kasiske B.L., Snyder J.J., Gilbertson D.T., Wang C.	856
25	Sirolimus (rapamycin)-based therapy in human renal transplantation: similar efficacy and different toxicity compared with cyclosporine	1999	Groth C.G., Bäckman L., Morales J.-M., Calne R., Kreis H., et al.	854
26	HLA-mismatched renal transplantation without maintenance immunosuppression	2008	Kawai T., Cosimi A.B., Spitzer T.R., Tolkoff-Rubin N., Suthanthiran M., et al.	850
27	Lymphomas after solid organ transplantation: a Collaborative Transplant Study report	2004	Opelz G., Döhler B.	850
28	Delayed graft function: risk factors and implications for renal allograft survival	1997	Ojo A.O., Wolfe R.A., Held P.J., Port F.K., Schmouder R.L.	835
29	Interleukin-2-receptor blockade with daclizumab to prevent acute rejection in renal transplantation	1998	Vincenti F., Kirkman R., Light S., Bumgardner G., Pescovitz M., et al.	821
30	Sirolimus for Kaposi's sarcoma in renal-transplant recipients	2005	Stallone G., Schena A., Infante B., Di Paolo S., Loverre A., et al.	819
31	Long-term consequences of kidney donation	2009	Ibrahim H.N., Foley R., Tan L., Rogers T., Bailey R.F., et al.	816
32	Polyomavirus-associated nephropathy in renal transplantation: interdisciplinary analyses and recommendations	2005	Hirsch H.H., Brennan D.C., Drachenberg C.B., Ginevri F., Gordon J., et al.	804
33	Poor long-term survival after acute myocardial infarction among patients on long-term dialysis	1998	Herzog C.A., Ma J.Z., Collins A.J.	801
34	Effect of fluvastatin on cardiac outcomes in renal transplant recipients: a multicentre, randomised, placebo-controlled trial	2003	Holdaas H., Fellström B., Jardine A.G., Holme I., Nyberg G., et al.	793
35	Delayed graft function in kidney transplantation	2004	Perico N., Cattaneo D., Sayegh M.H., Remuzzi G.	757
36	Acute kidney injury in patients hospitalized with COVID-19	2020	Hirsch J.S., Ng J.H., Ross D.W., Sharma P., Shah H.H., et al.	753
37	Costimulation blockade with belatacept in renal transplantation	2005	Vincenti F., Larsen C., Durrbach A., Wekerle T., Nashan B., et al.	746
38	Machine perfusion or cold storage in deceased-donor kidney transplantation	2009	Moers C., Smits J.M., Maathuis M.-H.J., Treckmann J., Van Gelder F., et al.	742
39	An assessment of the RIFLE criteria for acute renal failure in hospitalized patients	2006	Uchino S., Bellomo R., Goldsmith D., Bates S., Ronco C.	729
40	Clinical pharmacokinetics of mycophenolate mofetil	1998	Bullingham R.E.S., Nicholls A.J., Kamm B.R.	718
41	A phase III study of belatacept-based immunosuppression regimens versus cyclosporine in renal transplant recipients (BENEFIT Study)	2010	Vincenti F., Charpentier B., Vanrenterghem Y., Rostaing L., Bresnahan B., et al.	714
42	Evolution and clinical pathologic correlations of de novo donor-specific HLA antibody post kidney transplant	2012	Wiebe C., Gibson I.W., Blydt-Hansen T.D., Karpinski M., Ho J., et al.	713
43	A randomized clinical trial of OKT3 monoclonal antibody for acute rejection of cadaveric renal transplants	1985	Ortho Multicenter Transplant Study Group	710
44	Skin cancer in kidney and heart transplant recipients and different long-term immunosuppressive therapy regimens	1999	Jensen P., Hansen S., Moller B., Leivestad T., Pfeifer P., et al.	707
45	Categories of non-heart-beating donors	1995	Kootstra G., Daemen J.H.C., Oomen A.P.A.	692
46	Survival in recipients of marginal cadaveric donor kidneys compared with other recipients and wait-listed transplant candidates	2001	Ojo A.O., Hanson J.A., Meier-Kriesche H.-U., Okechukwu C.N., Wolfe R.A., et al.	691
47	Relative role of genetic complement abnormalities in sporadic and familial aHUS and their impact on clinical phenotype	2010	Noris M., Caprioli J., Bresin E., Mossali C., Pianetti G., et al.	686
48	Role of intestinal P-glycoprotein (mdr1) in interpatient variation in the oral bioavailability of cyclosporine	1997	Lown K.S., Mayo R.R., Leichtman A.B., Hsiao H.-L., Turgeon D.K., et al.	680
49	Long-term survival in renal transplant recipients with graft function	2000	Ojo A.O., Hanson J.A., Wolfe R.A., Leichtman A.B., Agodoa L.Y., Port F.K.	678
50	Long-term renal allograft survival in the United States: a critical reappraisal	2011	Lamb K.E., Lodhi S., Meier-Kriesche H.-U.	672
51	Angiotensin II type 1-receptor activating antibodies in renal-allograft rejection	2005	Dragun D., Müller D.N., Bräsen J.H., Fritsche L., Nieminen-Kelhä M., et al.	670
52	Randomised trial of basiliximab versus placebo for control of acute cellular rejection in renal allograft recipients	1997	Nashan B., Moore R., Amlot P., Schmidt A.-G., Abeywickrama K., Soulillou J.-P.	669
53	A comprehensive risk quantification score for deceased donor kidneys: the kidney donor risk index	2009	Rao P.S., Schaubel D.E., Guidinger M.K., Andreoni K.A., Wolfe R.A., et al.	666
54	Risk of end-stage renal disease following live kidney donation	2014	Muzaale A.D., Massie A.B., Wang M.-C., Montgomery R.A., McBride M.A., et al.	657
55	Kidney Disease: Improving Global Outcomes (KDIGO) anemia work group. KDIGO clinical practice guideline for anemia in chronic kidney disease	2012	McMurray J.J.V., Parfrey P.S., Adamson J.W., Aljama P., Berns J.S., et al.	654
56	Complement-binding anti-HLA antibodies and kidney-allograft survival	2013	Loupy A., Lefaucheur C., Vernerey D., Prugger C., Van Huyen J.-P.D., et al.	637
57	Risk factors for chronic rejection in renal allograft recipients	1993	Almond P.S., Matas A., Gillingham K., Dunn D.L., Payne W.D., et al.	636
58	Cardiovascular disease after renal transplantation	1996	Kasiske B.L., Guijarro C., Massy Z.A., Wiederkehr M.R., Ma J.Z.	634
59	Multicenter randomized trial comparing tacrolimus (FK506) and cyclosporine in the prevention of renal allograft rejection: a report of the European Tacrolimus Multicenter Renal Study Group	1997	Mayer A.D., Dmitrewski J., Squifflet J.-P., Besse T., Grabensee B., et al.	633
60	Donor characteristics associated with reduced graft survival: an approach to expanding the pool of kidney donors	2002	Port F.K., Bragg-Gresham J.L., Metzger R.A., Dykstra D.M., Gillespie B.W., et al.	624
61	Preexisting donor-specific HLA antibodies predict outcome in kidney transplantation	2010	Lefaucheur C., Loupy A., Hill G.S., Andrade J., Nochy D., et al.	620
62	Genetic polymorphisms of the CΥP3A4, CΥP3A5, and MDR-1 genes and pharmacokinetics of the calcineurin inhibitors cyclosporine and tacrolimus	2003	Hesselink D.A., Van Schaik R.H.N., Van Der Heiden I.P., Van Der Werf M., Smak Gregoor P.J.H., et al.	617
63	Identifying specific causes of kidney allograft loss	2009	El-Zoghby Z.M., Stegall M.D., Lager D.J., Kremers W.K., et al.	611
64	Comparison of survival probabilities for dialysis patients vs cadaveric renal transplant recipients	1993	Port F.K., Wolfe R.A., Mauger E.A., Berling D.P., Jiang K.	611
65	Incidence of non-Hodgkin lymphoma in kidney and heart transplant recipients	1993	Opelz G., Henderson R.	611
66	Post-transplant renal function in the first year predicts long-term kidney transplant survival	2002	Hariharan S., McBride M.A., Cherikh W.S., Tolleris C.B., Bresnahan B.A., Johnson C.P.	610
67	Waiting time on dialysis as the strongest modifiable risk factor for renal transplant outcomes: a paired donor kidney analysis	2002	Meier-Kriesche H.-U., Kaplan B.	605
68	Effects of hydroxyethylstarch and gelatin on renal function in severe sepsis: a multicentre randomised study	2001	Schortgen F., Lacherade J.-C., Bruneel F., Cattaneo I., Hemery F., et al.	599
69	Performance of the modification of diet in renal disease and Cockcroft-Gault equations in the estimation of GFR in health and in chronic kidney disease	2005	Poggio E.D., Wang X., Greene T., Van Lente F., Hall P.M.	593
70	Prevention of organ allograft rejection by a specific Janus kinase 3 inhibitor	2003	Changelian P.S., Flanagan M.E., Ball D.J., Kent C.R., Magnuson K.S., et al.	588
71	Rabbit antithymocyte globulin versus basiliximab in renal transplantation	2006	Brennan D.C., Daller J.A., Lake K.D., Cibrik D., Del Castillo D.	586
72	Pharmacokinetics and pharmacodynamics of systemically administered glucocorticoids	2005	Czock D., Keller F., Rasche F.M., Häussler U.	585
73	Bone marrow contributes to renal parenchymal turnover and regeneration	2001	Poulsom R., Forbes S.J., Hodivala-Dilke K., Ryan E., Wyles S., et al.	581
74	A worldwide, phase III, randomized, controlled, safety and efficacy study of a sirolimus/cyclosporine regimen for prevention of acute rejection in recipients of primary mismatched renal allografts	2001	MacDonald A.S.	581
75	Recommendations for the outpatient surveillance of renal transplant recipients	2000	Kasiske B.L., Vazquez M.A., Harmon W.E., Brown R.S., Danovitch G.M., et al.	578
76	20 years or more of follow-up of living kidney donors	1992	Najarian J.S., McHugh L.E., Matas A.J., Chavers B.M.	561
77	Incidence of BK with tacrolimus versus cyclosporine and impact of preemptive immunosuppression reduction	2005	Brennan D.C., Agha I., Bohl D.L., Schnitzler M.A., Hardinger K.L., et al.	554
78	Identification of a B cell signature associated with renal transplant tolerance in humans	2010	Newell K.A., Asare A., Kirk A.D., Gisler T.D., Bourcier K., et al.	552
79	Association of chronic kidney graft failure with recipient blood pressure	1998	Opelz G., Wujciak T., Ritz E.	550
80	Maintenance immunosuppression with target-of-rapamycin inhibitors is associated with a reduced incidence of de novo malignancies	2005	Kauffman H.M., Cherikh W.S., Cheng Y., Hanto D.W., Kahan B.D.	549
81	A randomized clinical trial of cyclosporine in cadaveric renal transplantation	1983	Reeve C.E., Harley F., Klassen J., Jeffery J., Halloran P., et al.	549
82	Perioperative mortality and long-term survival following live kidney donation	2010	Segev D.L., Muzaale A.D., Caffo B.S., Mehta S.H., Singer A.L., et al.	548
83	Rituximab and intravenous immune globulin for desensitization during renal transplantation	2008	Vo A.A., Lukovsky M., Toyoda M., Wang J., Reinsmoen N.L., et al.	544
84	A new arenavirus in a cluster of fatal transplant-associated diseases	2008	Palacios G., Druce J., Du L., Tran T., Birch C., et al.	544
85	Deceased-donor characteristics and the survival benefit of kidney transplantation	2005	Merion R.M., Ashby V.B., Wolfe R.A., Distant D.A., Hulbert-Shearon T.E., et al.	543
86	Improvement in outcomes of clinical islet transplantation: 1999-2010	2012	Barton F.B., Rickels M.R., Alejandro R., Hering B.J., Wease S., et al.	540
87	Long-term risks for kidney donors	2014	Mjøen G., Hallan S., Hartmann A., Foss A., Midtvedt K., et al.	537
88	Antibody-mediated microcirculation injury is the major cause of late kidney transplant failure	2009	Einecke G., Sis B., Reeve J., Mengel M., Campbell P.M., et al.	531
89	Association between delayed graft function and allograft and patient survival: a systematic review and meta-analysis	2009	Yarlagadda S.G., Coca S.G., Formica R.N., Poggio E.D., Parikh C.R.	531
90	Cyclosporine metabolism in human liver: Identification of a cytochrome P-450III gene family as the major cyclosporine-metabolizing enzyme explains interactions of cyclosporine with other drugs	1988	Kronbach T., Fischer V., Meyer U.A.	529
91	Cystatin C: an improved estimator of glomerular filtration rate?	2002	Laterza O.F., Price C.P., Scott M.G.	521
92	Plasmapheresis and intravenous immune globulin provides effective rescue therapy for refractory humoral rejection and allows kidneys to be successfully transplanted into cross-match-positive recipients	2000	Montgomery R.A., Zachary A.A., Racusen L.C., Leffell M.S., King K.E., et al.	521
93	Cancer risk after renal transplantation in the nordic countries, 1964-1986	1995	Birkeland S.A., Storm H.H., Lamm L.U., Barlow L., Blohmé I., et al.	517
94	Risk of cancer in renal-transplant recipients	1973	Hoover R., Fraumeni Jr. J.	516
95	Collaborative United Kingdom-Australasian study of cancer in patients treated with immunosuppressive drugs	1979	Kinlen L.J., Doll R., Sheil A.G.R.	515
96	Effect of waiting time on renal transplant outcome	2000	Meier-Kriesche H.-U., Port F.K., Ojo A.O., Rudich S.M., Hanson J.A., et al.	514
97	Expanded criteria donors for kidney transplantation	2003	Metzger R.A., Delmonico F.L., Feng S., Port F.K., Wynn J.J., Merion R.M.	512
98	Marked prolongation of porcine renal xenograft survival in baboons through the use of α1,3-galactosyltransferase gene-knockout donors and the cotransplantation of vascularized thymic tissue	2005	Yamada K., Yazawa K., Shimizu A., Iwanaga T., Hisashi Y., et al.	510
99	COVID-19 in solid organ transplant recipients: Initial report from the US epicenter	2020	Pereira M.R., Mohan S., Cohen D.J., Husain S.A., Dube G.K., et al.	503
100	Executive summary of the 2017 KDIGO Chronic Kidney Disease-Mineral and Bone Disorder (CKD-MBD) Guideline Update: what's changed and why it matters	2017	Ketteler M.; Block G.A.; Evenepoel P.; Fukagawa M.; Herzog C.A.; et al.	501
